# Ginger: a representative material of herb-derived exosome-like nanoparticles

**DOI:** 10.3389/fnut.2023.1223349

**Published:** 2023-07-13

**Authors:** He Zhu, Wenxi He

**Affiliations:** ^1^Department of Pharmacy, Tongji Hospital, Tongji Medical College, Huazhong University of Science and Technology, Wuhan, Hubei, China; ^2^NHC Key Laboratory of Respiratory Diseases, the Center for Biomedical Research, Tongji Hospital, Tongji Medical College, Huazhong University of Science and Technology, Wuhan, Hubei, China

**Keywords:** ginger, exosome, nanoparticle, herb, traditional Chinese medicine

## Abstract

Edible plant-derived exosome-like nanoparticles (PELNs) provide numerous benefits, including high yield, low cost, ethical compatibility, and multiple health benefits, which enable them to address technical constraints associated with mammalian nanoparticles. Herbs, known for their abundant bioactive components, are considered the primary source of natural medicines within the plant kingdom. Recently, a number of herbaceous sources have been investigated for the isolation and functionality of exosome-like nanoparticles (ELNs). However, they are commonly referred to as PELNs, and their distinct pharmacological properties are overlooked. In this review, these herb-derived ELNs are designated as HELNs, a novel herbal product that may also exhibit superior pharmacological activity compared to other types of PELNs. Among the documented HELNs, ginger-derived exosome-like nanoparticles (GELNs) are the most extensively studied. This review employs GELNs as an exemplar to delineate the process of extraction and purification, together with their physical and biochemical characteristics and therapeutic potential. The aim of this review is to promote the development and application of HELNs, and future research is encouraged to uncover their additional properties, extending beyond those of GELNs.

## 1. Introduction

### 1.1. Extracellular vesicles

Extracellular vesicles (EVs) are membrane-bound nano-size vesicles containing lipids, proteins, and nucleic acids that are released by virtually all living cells (1, 2). They have been considered as a means of long-distances communication between cells in different regions (3). Since their discovery over 50 years ago in mammalian plasma (4), EVs have been extensively studied in various scientific fields, including nutrition (5), nanotechnology for drug delivery (6), and clinical diagnostics and therapies (7, 8), resulting in the elucidation of their characteristics, functions, and underlying mechanisms. Based on the cellular biogenesis and size, mammalian EVs (MEVs) can be generally divided into exosomes (30–150 nm), microvesicles (100–1,000 nm), and apoptotic bodies (>1,000 nm). Nowadays, various feature, extraction, and application of MEVs have been already reported (3, 9) and are not repeated in this review.

### 1.2. Plant-derived exosome-like nanoparticles

Plants also contains EVs, which were first isolated from sunflower seeds by Regemte et al. (10) by vacuum infiltration-centrifugation method in 2009. Since then, scholars have successively isolated EVs from different plants and found that they have biomolecular structures similar to the mammalian exosomes in aspects of their size (50–200 nm) and biological cargo (lipid, protein, and small RNA), and are collectively named exosome-like nanoparticles (ELNs) (11, 12). At present, the characterization and function of ELNs from various plants (PELNs), such as grapefruits (13–15), grapes (14, 16, 17), carrots (14), and broccoli (18, 19) have been described. They have surprisingly been shown to possess cross-kingdom health benefits, including anti-oxidant (20, 21), anti-inflammatory (16, 22–24), anti-cancer (25–27), and antibiotic effects (28, 29). Furthermore, PELNs have more readily available raw materials than MEVs. For example, Li et al. (30) described that they could process up to 3 liters of ginger juice as a starting material in 1 h using a normal juice blender, equivalent to the volume of 300 cell culture dishes (150 mm), and eventually obtain a PELNs yield of 3 × 10^12^ particles/L juices. In addition, nanotechnology-based drug delivery systems have emerged as an attractive strategy. Compared to MEVs and synthetic nanoparticles, PELNs are non-toxic properties, low immunogenicity, high bioavailability, and easy absorption, which helps to solve the poor bioavailability of many active ingredients (31). Although these superior characteristics of PELNs have been reviewed in previous publications (11, 20, 22, 25, 32), there are still many issues for researchers to refine and investigate.

### 1.3. Herb-derived exosome-like nanoparticles

The term “plant” encompasses a broad range of organisms, including woody plants, herbs, and lianas. Among them, herbs have been considered as the main source of traditional medicines for effective drugs because of the rich bioactive components (33–35). So far, numbers herbaceous sources have been investigated for the isolation of ELNs, including ginseng (36), sunflower (37), green tea (27, 38), *pueraria lobata* (24), yam (39), *Catharanthus roseus* (40), and ginger (14, 15, 20, 22, 23, 25, 28–30, 41–51). However, they are collectively referred to as PELNs and their unique pharmacological properties are ignored. In this review, these herb-derived ELNs are named HELNs. As a novel herbal product, HELNs may have superior pharmacological activity than other kinds of PELNs. Indeed, a recent study list evidence that ginger (a traditional herbal medicine)-derived ELNs (GELNs) possess stronger pharmacological activity than fruit- or vegetable-derived ELNs. It showed that GELNs have significant antioxidant and anti-inflammatory effects on macrophages, while grape-, grapefruit-, and carrot-derived ELNs lack such effects (14). However, the biogenesis, characterization, and application of HELNs remaining poorly understood.

Therefore, in this review, we focused on GELNs as a representative of HELNs to summary their extraction, purification, physical and biochemical characteristics, and application. This review aims to offer a comprehensive guide for HELNs and provide new insights into effective pharmacological strategies for herbal medicine.

## 2. Ginger and GELNs

Ginger rhizome (*Zingiber officinale Roscoe*) is a fresh root of a perennial herb in the *Zingiberaceae* family. It has been in used for centuries in many countries in Asia, such as India, Arabia, Africa, and China for treating a variety of conditions, such as headaches, nausea, colds, arthritis, rheumatism, muscle discomfort, and inflammation (52, 53). Ginger comprises a wide range of compounds, including fatty acid (3–6%), protein (9%,) carbs (60–70%), crude fiber (3–8%), ash (~8%), water (9–12%), and volatile oil (2–3%) (53, 54). Moreover, its spicy flavor and therapeutic properties are attributed to its major constituents, Gingerol (GN) and shogaol (SG) (52, 55).

**Figure 1:**
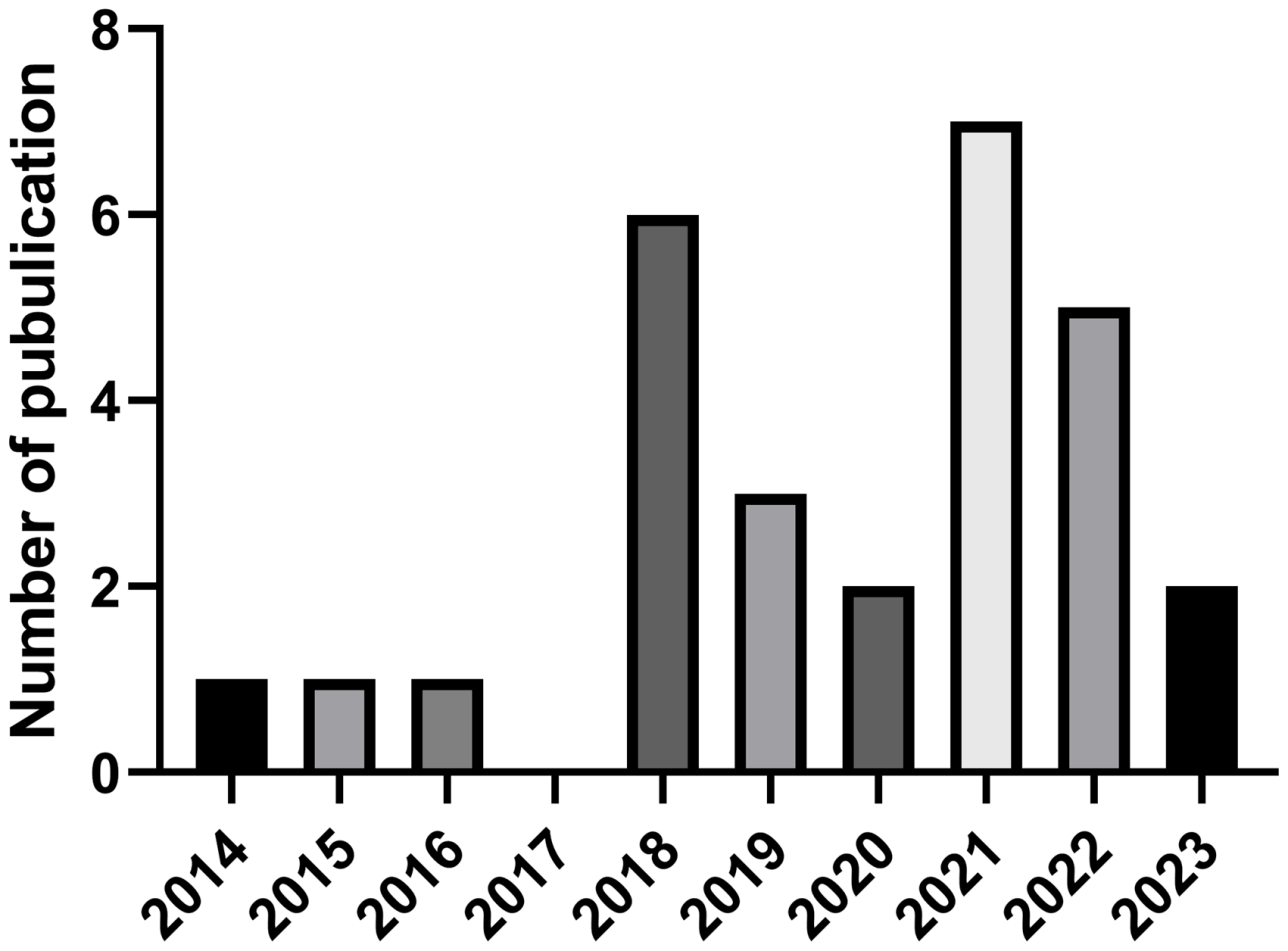
The number of GELNs-related global SCI journal articles varies over time. Data were obtained from Web of Science data base (Up to 03/10/2023). GELNs, ginger-derived exosome-like nanoparticles. SCI, Science Citation Index.

The initial isolation and description of GELNs was done by Huang-Ge Zhang’s laboratory in 2014 (14). Since then, about 28 studies related to GELNs have been published (Figure 1) (14, 15, 20, 22, 23, 25, 28–30, 41–51, 56–60). Researchers have employed various techniques to isolate and characterize GELNs, and evaluated their effectiveness in addressing different therapeutic objectives, including gut microbiota regulation (29, 44), anti-biosis (51, 61), anti-alcoholic liver damage (48), anti-inflammatory (22, 23, 25), anti-cancer (25), anti-SARS-COV-2 (15, 42), and prevention of type 2 diabetes (20, 44). GELNs is also a reliable and versatile carrier platform for delivering small molecular drugs and small RNAs (30, 43, 46, 47, 49, 50).

## 3. GELNs extraction

**Figure 2:**
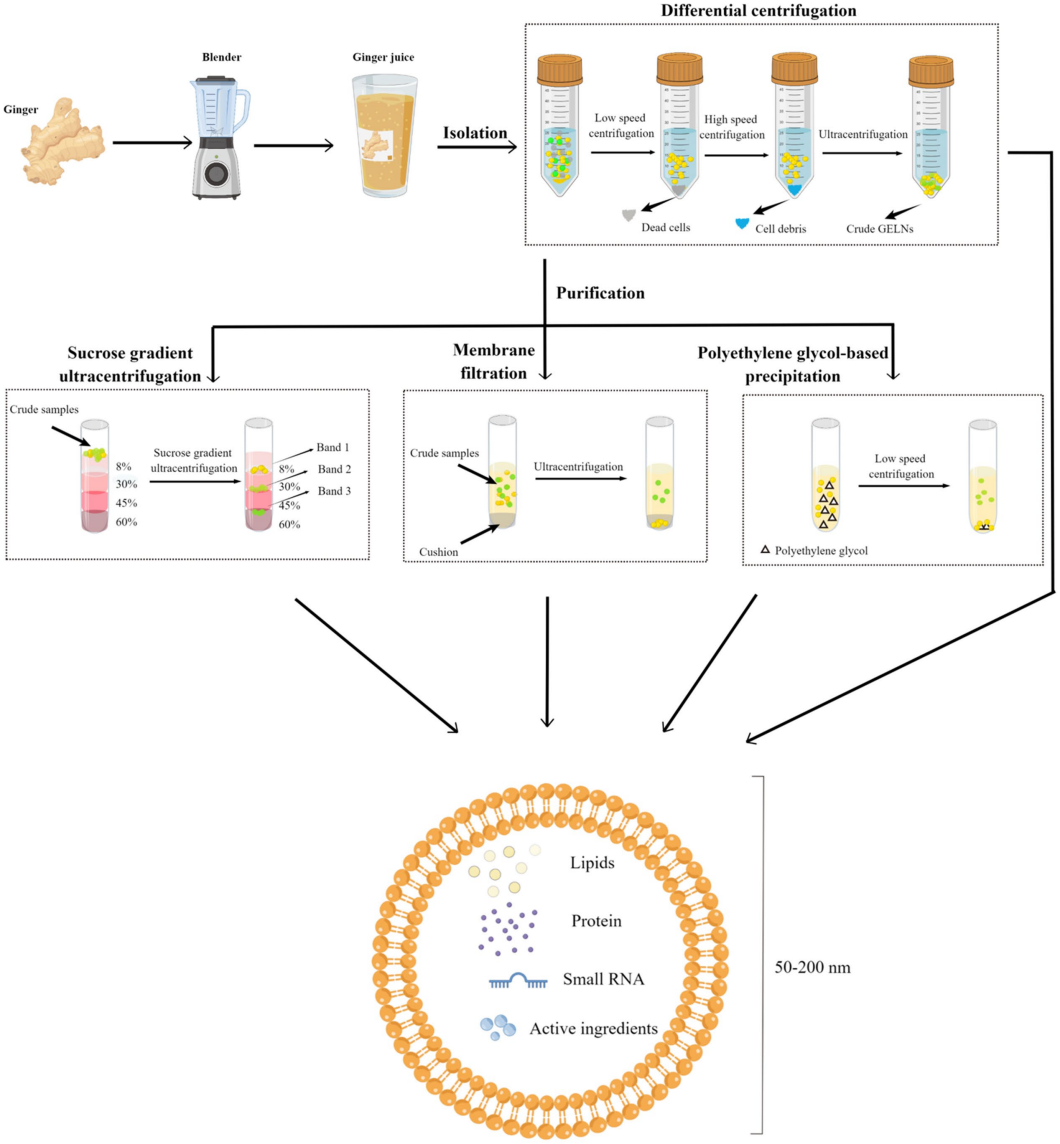
Schematic diagram of the GELNs isolation and purification methods. Fresh ginger was first processed using a breaker or blender to obtain ginger juice, and the juice was subjected to differential centrifugation to obtain crude GELNs, followed by a further purification to obtain pure GELNs. Currently reported methods for GELNs purification include sucrose gradient ultracentrifugation, membrane filtration, and PEG-based precipitation. The obtained GELNs showed a phospholipid bilayer structure of 50–200 nm, containing lipids, protein, small RNA, and active ingredients. GELNs, ginger-derived exosome-like nanoparticles. PEG, polyethylene glycol.

GELNs can be extracted from ginger using similar concepts and approaches used for MEVs, with some slight differences. Due to the relatively firm texture of ginger, a wall breaker or blender must first be used to extract the crude GELNs from their intra- or extracellular environment. Following this, differential centrifugation (DC) is the most commonly used and fundamental method for GELNs elementary extraction (45, 62). To obtain greater purity and better morphology, most studies have also utilized purification methods, including sucrose gradient ultracentrifugation (sgUC), membrane filtration (MF), PEG-based precipitation, and commercial kits (Table 1). Each of these technologies has advantages and disadvantages (Table 2). In this section, we summarized and discussed the reported methods for GELNs extraction and purification (Figure 2).

**Table 1:** Different methods for GELNs extraction.

Extraction method	Quantification	Yield	Year	References
DC only	N.R.	N.R.	2021	(50)
	N.R.	N.R.	2021	(51)
DC and sgUC	Protein	17.5 mg/kg tissue	2021	(43)
		0.9 mg/g tissue	2018	(29)
		50 mg/kg tissue	2016	(25)
		GDN, 3.79 ± 27 mg/g ginger protein	2015	(48)
		N.R.	2014	(14)
	Phosphorus	N.R.	2017	(46)
		48.5 ± 4.8 mg/kg tissue	2016	(47)
	Number of nanoparticles	1 × 10^12^ GDNP/g tissue	2022	(20)
		N.R.	2021	(42)
		5 × 10^12^ particles/g tissue	2021	(44)
		0.5 to 2 × 10^11^ vesicles/g tissue	2019	(23)
		N.R.	2019	(61)
MF and sgUC	Protein	3 × 10^12^ particles/L root juices	2018	(30)
DC, PEG-based precipitation and dialysis	The weight of the lyophilized powder	2–3.8 g/kg of tissue	2020	(45)
		Up to 5-fold, in pH 4 and pH 5	2021	(41)
DC and isolation kit	Protein	N.R.	2022	(22)

GELNs, ginger-derived exosome-like nanoparticles; DC, differential centrifugation; sgUC, sucrose gradient ultracentrifugation; MF, membrane filtration; PEG, Polyethylene glycol; N.R., not reported.

**Table 2:** Main advantages and disadvantages of the currently available methods for GELNs extraction.

Method	Advantages	Disadvantages	References
Differential centrifugation	Reduced cost and contamination risks; Large sample capacity	High equipment required, time consuming, damage the integrity ELNs	(50, 51, 63, 64)
Sucrose gradient ultracentrifugation	Pure preparations; absence of additional chemicals	Complexity, loss of sample; Virus particles cannot be distinguished	(65, 66)
Membrane filtration	High purity; Easy to operate	Membrane contamination, products loss due to attaching to the membranes	(65, 67)
PEG-based precipitation	Easy to operate; maintains EV integrity; no additional equipment required	Contamination and retention of the polymer	(41, 45, 67)
Commercial kits	Easy to operate	Cost; Low purity; Unknown separation mechanism	(22)

### 3.1. GELNs extraction using differential centrifugation

The most widely used method for GELNs extraction is DC, which involves a low speed (3,000 g for 20 min) followed by a high speed (10,000 g for 40 min) to remove cells and large debris, with ultracentrifugation performed at 150,000 g for 2 h to precipitate the ELNs (68). Although DC is effective, there can be variations in the size and zeta potential values of the GELNs obtained using this method. For instance, Man et al. (50) consistently produced GELNs whose average size was 70.09 ± 19.24 nm with a mean zeta potential of −27.70 ± 12.20 mV using this method. Additionally, Adb El Wahab et al. (51) changed the ultracentrifugation strategy from a single 2 h to 1 h twice, and obtained GELNs with an average size of 238.3 nm and a zeta potential value of −13 mV using the DC method. The large differences between the GELNs obtained with these two similar methods suggest that DC may be crude and unstable. Further studies are needed to validate this speculation. Nonetheless, GELNs extracted via the DC method are generally acceptable for pharmacological studies despite low purity levels (50, 51).

### 3.2. The purification of GELNs using sgUC

The sgUC method is widely used due to its cost-effectiveness and ease of implementation (Table 1). Following extraction by DC, the crude GELNs were subjected to sucrose gradient centrifugation using a combination of different sucrose concentrations (8, 15, 30, 45, and 60%) at a speed of 150,000 g for 2 h (Figure 2). GELNs located in bands 1 (8/30% interface) and 2 (30/45% interface) were collected together or separately for downstream experimentation, while those in band 3 (45/60% interface) were discarded due to their low yield and instability (Figure 2). The GELNs found in different bands were named as GELN1, GELN2, and GELN3, respectively. Their respective sizes were 292.5, 231.6, and 219.6 nm (25) (Table 3). Following quantification, the yield of GELNs was found to be in the range of 20–100 mg/kg ginger tissue (by bicinchoninic acid assay) (25, 29, 48, 62) or 0.2–1 × 10^12^ particles/g tissue (by nanoparticle tracking analysis) (20, 23). Didier Merlin and his colleagues publicly presented the detailed protocol for DC and sgUC in the journal *BIO-PROTOCOL* (58).

**Table 3:** Technologies used for the detection of physical characteristics of GELNs.

Technology	Equipment	GELNs size (nm in diameter)	Zeta potential (mv)	Year	References
NTA	NanoSight NS300	~200*	N.R.	2021	(44)
		~200*	N.R.	2019	(61)
		132	N.R.	2019	(23)
		110.5 ± 27.49	N.R.	2018	(30)
DLS	Zetasizer Nano S90	250 ± 72	−220 ± 131	2022	(20)
		156 ± 36	−26.6 ± 5	2022	(22)
		~232.7	−18.1	2017	(46)
		~188.5	−20	2016	(47)
		GELN1: 386.6 GELN2: 294.1	GELN1: −24.6 GELN2: −29.7	2015	(48)
	Zetasizer Nano ZS	70.09 ± 19.24	−27.70 ± 12.20	2021	(50)
		PH4: 294.1 PH5: 343.8 PH6: 422.7 PH7: 518.2 PH8: 519.2 PH9: 545.9	PH4: −31.9 PH5: −32.2 PH6: −28 PH7: −27.8 PH8: −27.3 PH9: −24.2	2021	(41)
		180.5 ± 69.7	N.R.	2021	(42)
		~250	−30	2014	(14)
		Ultra: 403 15%PEG: 252	Ultra: −25.7 15%PEG: −21.2	2020	(45)
		206.8 ± 81.1	N.R.	2018	(29)
	ZetaPlus instrument	238.3	−13	2021	(51)
		GELN1: 292.5 GELN2: 231.6 GELN3: 219.6	GELN1 and GELN2: −12 GELN3: −2.1	2016	(25)
	Nicomp 380	~ 284.6	−12.1	2021	(43)

GELNs, ginger-derived exosome-like nanoparticles; N.R., no reported.

### 3.3. The purification of GELNs using MF method

Although sgUC is widely used to purify GELNs, it has limitations as the high-speed centrifugal force can impair the integrity of the ELNs or provoke their aggregation, making the process inefficient and time-consuming (69, 70). Li et al. (30) have recently addressed this issue by utilizing a thin OptiprepTM cushion (60% iodixanol) at the bottom of the centrifuge tube to avoid damage and aggregation in DC-extracted crude GELNs. Thus, the MF method improved not only the yield but also the quality of GELNs. They found that MF method yielded more GELNs (5.76 μg/mL root juices) than the DC method (1.78 μg/mL) (30). According to nanoparticle tracking analysis (NTA), there was no significant difference in the size of GELNs between these two methods. In addition, MF-purified GELNs had a cleaner background, a nicer spherical shape, and less aggregation than those extracted using the DC method (30). However, this method still relies on ultracentrifugal equipment, making large-scale production difficult.

### 3.4. Polyethylene glycol-based purification for GELNs

PEG-based precipitation represents an alternative method for purifying GELNs that can efficiently boost their production without the use of ultracentrifugation (45). This technique described involves the use of an aqueous PEG, a bioaffinity network-structured polymer, to encapsulate EVs prior to precipitation (71). It leads to the formation of EVs aggregates that can be precipitated by low-speed centrifugation at 1,500 g (65). Although the isolated EVs are in the same size range as other common isolation techniques like sgUC, the PEG-based EVs purification procedure causes a decrease in their purity and specificity by co-precipitating soluble non-exosomal proteins. As a consequence, the final pellet from this method contains a mixture of non-exosomal proteins, immunoglobulins, viral particles, immune complexes, and other contaminants (65, 68).

Previous studies have effectively and extensively employed PEG to capture MEVs (72, 73). Due to the similarity in size and biological structure with MEVs, Kalarikkal et al. (45) attempted a PEG-based precipitation method to purify GELNs. Firstly, the ginger was peeled and juiced. Next, the obtained fresh juice was centrifuged at 2,000 g for 10 min, 6,000 g for 20 min, and 10,000 g for 45 min to remove large fibers/cells, large particles/cell debris, and microparticles, respectively. Then, the supernatant obtained after the centrifugation of 10,000 g step was mixed with different concentrations of PEG and incubated at 4°C overnight with gentle rocking. Finally, the mixture was centrifuged at 8,000 g for 30 min at 4°C to obtain purified GELNs. As a result, they found that PEG6000 produced more GELNs than PEG4000 and PEG8000. Subsequently, by adjusting the concentration of PEG6000 (8, 10, 12, and 15%) during purification, they yielded GELNs with average sizes of 365 nm, 304 nm 263 nm, and 252 nm (45). Moreover, the average sizes of GELNs obtained using the PEG-based method were smaller than those of DC-extracted GELNs (403 nm). Meanwhile, the overall yield of GELNs when using the PEG6000 method was 2–3.8 g/kg ginger tissue and 4 g/kg when using the DC method. In addition, the PEG-based method for GELNs extraction was shown to be equivalent to DC in shape and the active ingredients, including small RNA, proteins, and lipids. The authors also compared the expenses of the PEG approach with the DC method and found that the PEG method is 5–10 times less expensive than the DC method (45).

In another study, Suresh et al. (41) improved the PEG-based precipitation method and revealed that adjusting the pH environment could boost the yield of GELNs. They adjusted PEG6000 at pH 5, extracting 4-5-fold higher GELNs than when using the DC method without affecting the main active components (41). Collectively, this new method has the potential to produce GELNs on a large scale and economically. However, this technique still requires further optimization to achieve higher purity and yield values, making it applicable to large-scale production.

### 3.5. Commercial kit-based purification for GELNs

In addition, only one study used a commercial kit for GELNs purification (22). They firstly employed a DC method to obtain crude GELNs. Then, the samples were mixed and incubated with commercial reagents, followed by centrifugation at 18,000 g for 60 min to obtain GELNs. Although the composition of commercial reagents is not disclosed, the kit may employ at least one of the techniques already discussed.

## 4. Physical characteristics of GELNs

There is a need to further assess the physical characteristics of GELNs obtained by the above methods, including particle size, zeta potential, and morphological structure, to determine their presence and integrity. The items and techniques currently used to detect characteristics of GELNs are derived from MEVs, and no unique detections have been developed. This section summarized the physical characteristics of GELNs and their conventional detection techniques.

### 4.1. Size distribution and zeta potential of GELNs

Size and zeta potential are essential characteristics of GELNs. They can provide information about the stability and distribution potential of GELNs (74). Previous studies show that GELNs are between 100 and 500 nm in size. The zeta potential of GELNs typically ranges from −10 to −30 mV, except for a single study that obtained a −220 ± 131 mV result (Table 3) (20). Zeta potential is an important value because it can signify the presence or absence of stable smaller particles (75). High absolute values of zeta potential show a more stable system of smaller dispersed particles, while lower ones indicate a higher tendency to coagulate or agglomerate (76). GELNs are nanoscale in size, so specialized detection techniques are required. Dynamic light scattering (DLS) and NTA are the main techniques used to detect their size distribution (Table 3). While DLS can detect zeta potential, NTA is not well suited for it (76). DLS is preferred over NTA due to the fact that it can detect both the size distribution and the zeta potential of GELNs.

### 4.2. Morphology of GELNs

The double-layered vesicle structure is a fundamental characteristic of GELNs. The morphology of GELNs has been studied using different detection techniques, including transmission electron microscopy (TEM) (14, 20, 22, 25, 29, 30, 42–44, 46, 47, 50, 58, 61), atomic force microscopy (AFM) (25, 46–49, 56), and scanning electron microscopy (SEM) (23). In addition, detailed protocols for TEM and AFM observation of GELNs were reported in the journal *BIO-PROTOCOL* by Didier Merlin and his colleagues (56, 58).

**Figure 3:**
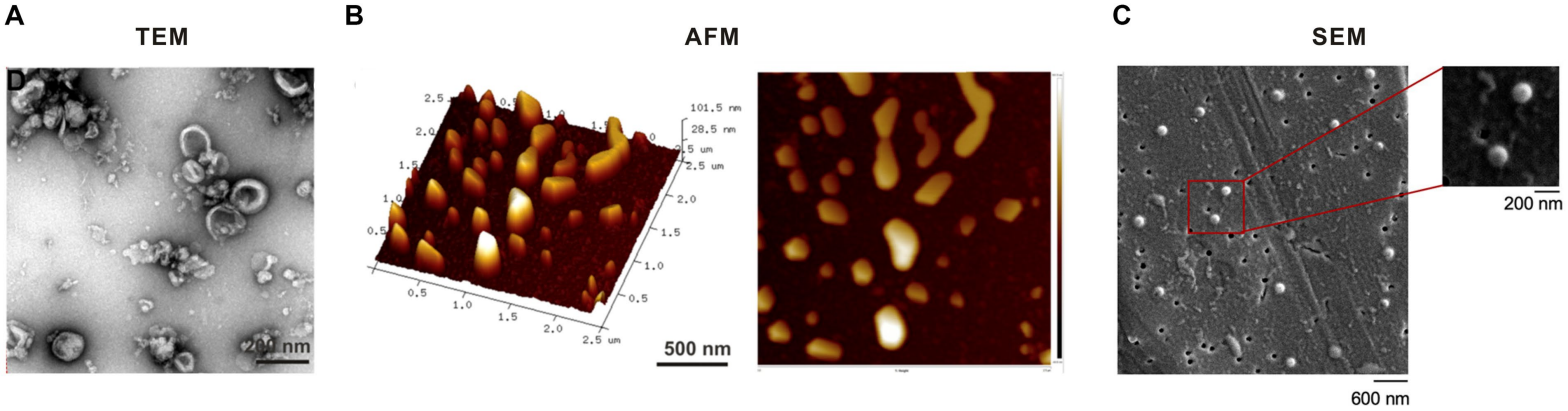
Morphological characterization of ginger-derived exosome-like nanoparticles by **(A)** transmission electron microscopy (TEM) (25), **(B)** atomic force microscopy (AFM) (25), and **(C)** scanning electron microscopy (SEM) (23).

TEM, which has a resolution of 0.1–0.2 nm and can magnify an object up to millions of times, is particularly well-suited for observing the bilayer vesicles. TEM imaging shows that GELNs share structural similarities with conventional exosomes, presenting a round, teacup-shaped morphology (58) (Figure 3A). AFM is a microscopy technique that utilizes a metallic “tip” to scan the sample surface thereby measuring its topology and properties. One of the advantages of AFM is its simple sample preparation which requires no conductive pretreatment. AFM images show that GELNs are nano-sized particles with a spherical shape and good size homogeneity (56) (Figure 3B). SEM is an advanced microscopic tool that provides morphological observations between Transmission Electron Microscopy (TEM) and an ordinary light microscope. SEM offers the advantage of simple sample preparation. The morphology of GELNs was investigated in a single study utilizing SEM. The study revealed that the particles exhibit sphere-shaped morphology with a diameter range of 120–150 nm (23) (Figure 3C).

## 5. Biochemical characteristics of GELNs

**Figure 4:**
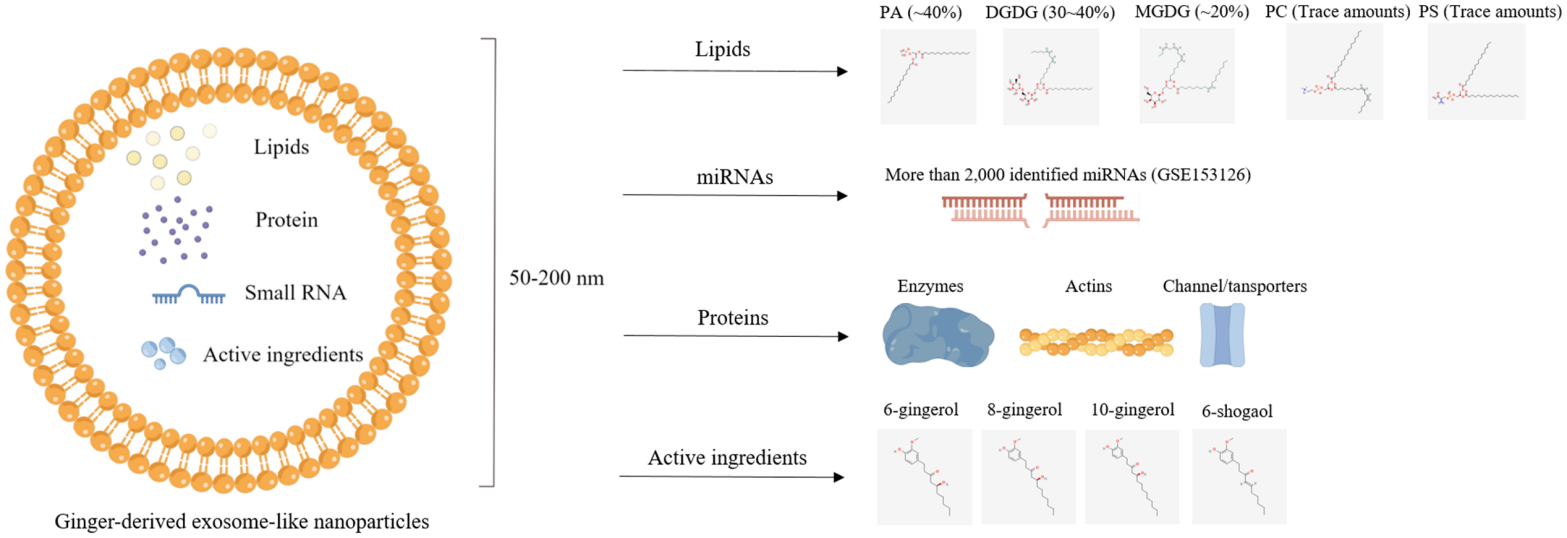
Illustration of bioactive components, including lipids, protein, miRNAs, and active ingredients, in ginger-derived exosome-like nanoparticles.

GELNs contain many bioactive components. One part is common to PELNs, including proteins, lipids, and small RNAs, while the other part comprises specific active ingredients distinctive for GELNs such as gingerols and shogaols (Figure 4) (52). In this section, we fully reviewed bioactive elements present in GELNs.

### 5.1. Lipids in GELNs

Generally, ELNs are spherical molecular assemblies composed of phospholipid bilayers (77). The phospholipid membranes maintain their structural stability (78), making the lipids the most abundant component of ELNs (79). Lipids in MEVs are mainly cholesterol and sphingomyelin (80, 81), while lipids in PELNs are mainly phospholipids, including phosphatidic acid (PA), phosphatidylethanolamine (PE), phosphatidylcholine (PC), phosphatidylserine (PS), and digalactosyldiacylglycerol (DGDG) (16, 62). More specifically, GELNs contained high proportions of PA (~40%) as well as DGDG (30 ~ 40%) and monogalactosyldiacylglycerol (MGDG) (~20%), whereas PC and PS were very low (25). Another study found a similar result that ELNs derived from turmeric (belonging to the ginger family) also showed high DGDG (~42%) and MGDG (~12%) along with PA (~20%) and PC (~16%) (82).

Lipids are the main components of the ELNs skeleton by affinity principle, enabling them to easily cross biological membranes to deliver drugs (83). For GELNs, Zhang et al. (25) found that shogaols encapsulated by GELNs could better target colonic epithelial cells and macrophages than the free-form shogaols that quickly pass through the intestine. Another recent study carried by Zhuang et al. (48) found that additional PE promoted the GELNs uptake by A549 cells, whereas PA and PC inhibited GELNs uptake.

More surprisingly, lipids in GELNs exhibit pharmacological effects. Chen et al. (23) showed that the Folch method (84) was used to extract lipids from GELNs, which proved to be active biomolecules that hinder the assembly and activation of the NLRP3 inflammasome in macrophages. Sundaram et al. (61) removed PA from GELNs using Thin layer chromatography (TLC) and showed that it was PA could directly bind to HBP35 protein in *P. gingivalis*, resulting in an antibacterial effect. Moreover, Kumar et al. (20) extracted lipid from the sucrose purified/washed band of processed GELNs and analysis it through lipidomic analysis with mass spectrometry. Their results showed that PA, a component of GELNs, induced Foxa2 expression in intestinal epithelial cells, altering the lipid composition of intestinal epithelial exosomes and preventing glucose intolerance and insulin resistance induced by the high-fat diet (20). Collectively, scientists previously focused on the transmembrane capacity of lipid bilayer structures, while recent pharmacological studies on lipids have opened new ideas.

### 5.2. miRNAs in GELNs

The miRNAs are small, noncoding RNAs whose primary function is to modulate gene expression by regulating mRNA cleavage or repressing translation (85). A previous study showed that miRNA cargo is more enriched in GELNs, while ginger tissue is rich in tRNAs (42). The popularity of miRNA deep sequencing technology has greatly facilitated researchers to investigate the types of miRNAs (86). Several studies have employed this technology and annotated miRNAs in GELNs. Zhang et al. (25) found 125 different miRNAs in GELNs, each containing between 15 and 27 nucleotides. These miRNAs could potentially target and regulate the expression of human genes by binding to their 3′-untranslated regions (3′-UTRs) using Targetscan, an online software for predicting miRNA targets. Similarly, another study identified 185 miRNAs in ginger tissue and GELNs. Among these miRNAs, there were 50 differentially expressed miRNAs in GELNs, including 21 upregulated and 29 downregulated, when compared with ginger tissue. Among these upregulated expressed miRNAs, the expression of bdi-miR5179, csi-miR396e-5p, and ptc-miR396g-5p was identified with ≥4-fold change (22). In addition, a prominent high-throughput profiling of miRNAs in GELNs carried out by Zhang’s lab identified 2,228 miRNAs, including 532 higher and 1,280 lower in GELNs compared to ginger tissue (GSE153126) (42). These miRNA sequencing data provide a great convenience for subsequent functional research of GELNs.

### 5.3. Protein in GELNs

Surface maker proteins, such as CD63, CD9, and CD81, and functional proteins have been identified in MEVs (87). However, research on protein in PELNs rarely explored. Only one previous study found a low protein content in GELNs using HPLC/MS, predominantly cytosolic protein (actin and proteolysis enzymes) and some membrane channel/transporters (aquaporin and chloride channels) (25). This observation is consistent with the protein profile in grape nanoparticles (16).

In addition, most studies on GELNs have detected and quantified obtained GELNs using the Bio-barcode amplification assay (BCA) method (Table 1). However, this method is unstable and temporary because the protein concentration varies with the extraction method, such as protein content increased in GELNs extracted under a low-pH setting (41).

### 5.4. Unique active ingredients in GELNs

Numerous studies based on clinical trials and animal models have shown the essential role of ginger and its active ingredients in treating diseases (88, 89). Phenolic compounds, including gingerols and shogaols, are mainly components (88). Studies reveled that GELNs also enriched with a wealth of phenolic compounds. The 6-gingerol, 8-gingerol, 10-gingerol, and 6-shogaol content in GELNs was 10.21-fold, 22.69-fold, and 32.36-fold higher than ginger slices by using HPLC (50). Another study used a triple quadrupole mass spectrometer and found that 6-gingerol and 6-shogaol in GELNs band 1 (8/30% after purification by sgUC) were 0.56 μg/mg and 0.22 μg/mg, respectively, and in GELNs band 2 (30/45%) were 5.68 μg/mg and 2.95 μg/mg, respectively (25). The following *in vivo* study demonstrated that GELNs band 2 with enriched 6-gingerol and 6-shogaol exerts better anti-inflammatory effects than in GELNs band 1 (25). Overall, extraction technology for ELNs is considered an active compound concentration technique. High concentrations of active compounds are enriched in ELNs and then transported to the target site. Therefore, it remains to be explored whether or not the concentration efficiency and compound composition changes due to ELNs extraction technology have any benefits.

## 6. Stability of GELNs

The stability of GELNs is a critical factor in their production, storage, and transport. GELNs have a phospholipid bilayer structure similar to mammalian cells and are sensitive to temperature, pH, and osmolarity, among other factors. Zhang et al. (47) found that GELNs were extremely stable when stored at 4°C for up to 25 days, as their size and zeta potential remained unchanged. Moreover, Chen et al. (23) found that freshly extracted GELNs and frozen GELNs stored at −80°C similarly inhibited IL-1β secretion and Casp1 autocleavage. However, the suitability of GELNs for long-term storage in liquid nitrogen requires further investigation, particularly regarding cryopreservation solutions and freezing conditions.

In addition, the physiological stability of GELNs is crucial in determining their bioavailability, drug delivery, and targeting effects. Since oral administration is the most common and appropriate method for GELN application, its *in vivo* stability is of utmost importance. Zhuang et al. (48) simulated *in vivo* conditions by exposing GELNs to a stomach-like solution (pH 2.0) and a small intestine-like solution (pH 6.5) to test their stability under physiological conditions. The authors observed that GELNs inflated in the stomach-like solution and further distended in the small intestine-like solution. Additionally, the surface charge of GELNs shifted from negative to positive in stomach-like solution, while it returned to negative in small intestinal-like solution (48). In a separate study, Zhang et al. (25) tested the stability of GELNs *in vitro* by subjecting them to pH 2.0 to 6.5 solutions at 37°C. They observed a slight reduction in GELN size in both stomach- and intestine-like solutions, which was inconsistent with the study reported by Zhuang et al. (48). Meanwhile, the zeta potential of GELNs changed based on the pH value. In a near-neutral pH solution (phosphate buffered saline), and an intestine-like solution, the surface of GELNs had negatively charged (−14.2 mV and − 7.3 mV, respectively), while it had a week positive charge (0.26 mV) in an acidic stomach-like solution (25). Importantly, these findings are consistent with earlier studies that acidic condition is a suitable environment for the existence of exosomes (41).

## 7. Therapeutic benefits of GELNs

**Figure 5:**
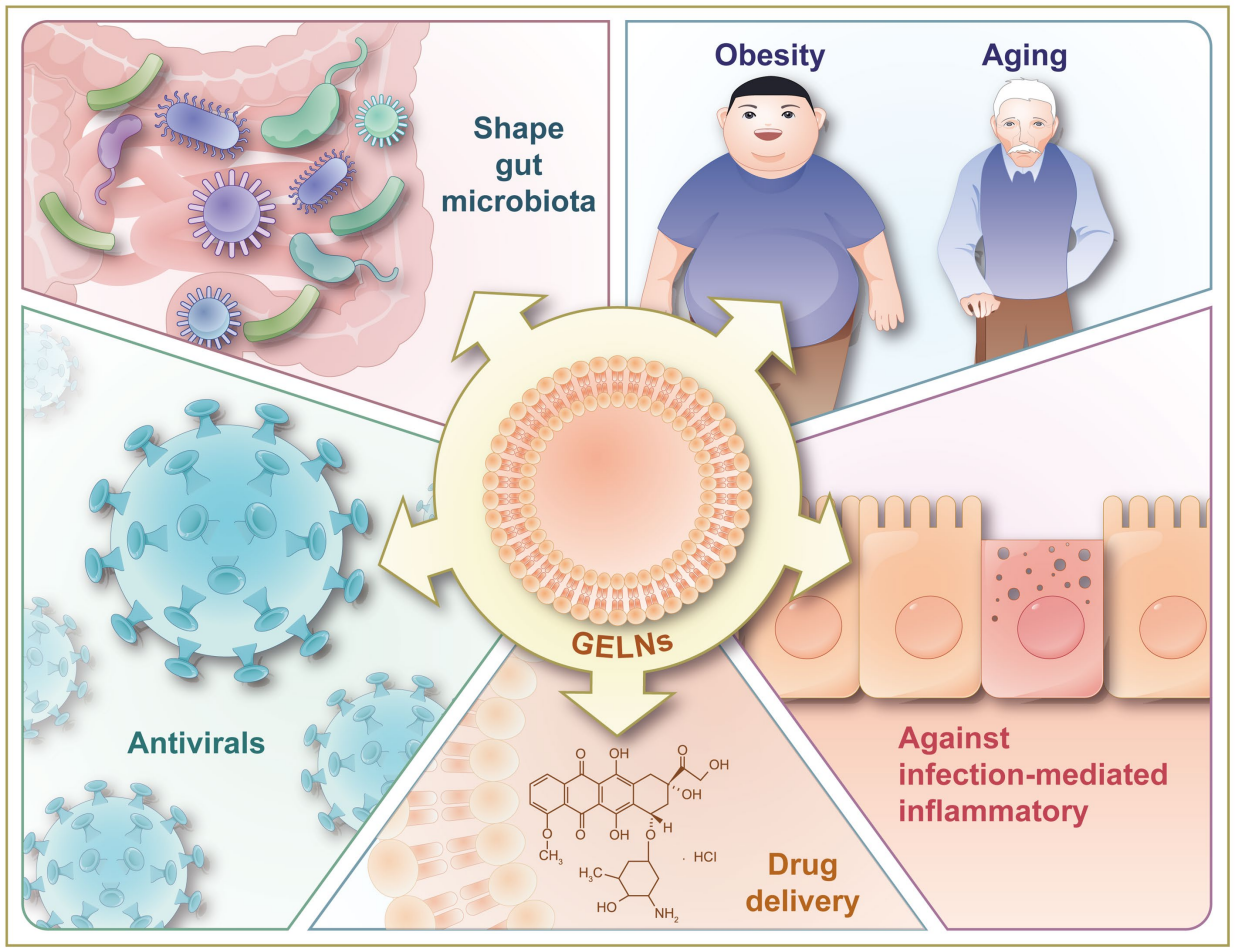
Illustration of therapeutic benefits of GELNs, including against inflammation-related chronic diseases (obesity and aging), against infection-mediated inflammatory, shaping gut microbiota, and antivirals. GELNs, ginger-derived exosome-like nanoparticles.

GELNs have shown promising therapeutic benefits, including anti-inflammatory (14, 22, 23, 25, 44), anti-cancer (25, 47), anti-bacterial (29, 51, 61), anti-viral (15, 41, 42), and drug delivery effects (30, 46, 50, 57) (Figure 5). This section summarizes the latest applications of GELNs in order to inspire future studies.

### 7.1. Anti-inflammatory effects

Inflammation is the predominant defensive reaction to heterogeneous stimuli in the body, manifested by swelling, heat, pain, and dysfunction (90). Excessive inflammation also facilitates the development of autoimmune diseases. Ginger preparations are well-recognized traditional medicine for stabilizing immune homeostasis (91, 92). Especially, ginger extracts, including 6-poradol, 6-shogaol, 6-gingerol, 8-gingerol, and 10-gingerol have shown considerable anti-inflammatory activity (93). For example, a previous study showed that 6-gingerol inhibits reactive oxygen species (ROS) levels and Inducible NO Synthase (iNOS) expression by reducing Nuclear Factor Kappa B (NF-kB) activation and protein kinase C (PKC) translocation (94). In addition, 6-gingerol inhibits cytokine production and T cell activation, preventing or alleviating allergic rhinitis in a mouse model of allergy symptoms (95). Interestingly, recent studies have also found that GELNs possess an outstanding anti-inflammatory effect, particularly prominent in treating inflammation-related chronic diseases, such as obesity (20) and inflammatory bowel disease, and inflammatory response to infection, such as viral and bacterial infections.

#### 7.1.1. GELNs against inflammation-related chronic diseases

Macrophages are critical participants in chronic diseases and have the ability to release either anti-inflammatory or pro-inflammatory factors (96). According to Mu et al. (14), GELNs were found to selectively stimulate macrophages to produce the anti-inflammatory cytokine IL-10 and activate nuclear factor (erythroid-derived 2)-like 2 (Nrf2), leading to the attenuation of inflammatory effects. In addition, NLRP3 inflammasome regulates proinflammatory cytokines and instigates many chronic inflammatory diseases, such as obesity, aging, and inflammatory bowel disease (97). In a subsequent study, lipids within GELNs were identified as the active biomolecules that inhibit the assembly and activation of the NLRP3 inflammasome in macrophages. This was achieved using heat treatment to denature proteins and RNase to deplete RNAs (23).

Obesity and aging can lead to chronic low-grade inflammation in multiple tissues (98–100). Treating mice with GELNs not only protects them from chronic inflammation caused by high-fat diet (HFD) but also improves their lifespan (20). GELNs treatment inhibited the HFD-induced increase in pro-inflammatory cytokines, including IL-1β, TNF-α, and IL-6, and induced the anti-inflammatory cytokine (IL-10) in plasma. The cytokine reduction can be explained by low immune cell infiltration (F4/80 and CD3) noted in HFD-fed mice treated with GELNs compared to those treated with PBS (20). However, they just showed this phenotype at the end of the article without an in-depth underlying mechanism.

Inflammatory bowel diseases, including ulcerative colitis and Crohn, are chronic and debilitating inflammatory diseases (101, 102). As previously mentioned in 5.4, GELNs were enriched with active ingredients and possess an excellent therapeutic effect in reducing acute inflammation induced by dextran sodium sulfate (25). In addition, miRNAs encapsulated in GELNs play a very important role in the anti-inflammatory effect. Yin et al. (22) found that miRNAs within GELNs regulate inflammation in intestine cells challenged with LPS. It was shown that GELNs treatment significantly inhibited LPS-induced NF-κB overexpression and related inflammatory cytokines (22). However, this inhibitory effect was relieved when GELNs were pre-treated with RNase.

#### 7.1.2. GELNs against infection-mediated inflammatory

Bacterial infection initiates a cascade of innate immune responses (103). Many studies found that GELNs exert excellent immunomodulatory ability in infection. For example, infection by *P. gingivalis* enhanced the infiltration of T cells, macrophages, and leukocytes in periodontal tissues, which can be significantly reduced by GELNs treatment (61). Meanwhile, qPCR results indicated that GELNs significantly reduced *P. gingivalis*, induced pro-inflammatory cytokines, and documented bone resorptive cytokines, such as TNF-a, IL-1a, IL-1b, INF-g, IL-6, IL-13, and IL-22 (61). Another study found that GELNs can be absorbed by the bacteria in the mouse intestine (29). Moreover, the miRNAs within GELNs enhance inflammation by modifying the microbial composition (29). More interestingly, the authors also found that lipids in GELNs can signal to promote the uptake of GELNs by *Lactobacillus rhamnosus* (29). The mdo-miR-7267-3p, transported by GELNs, regulates the mRNA expression of *ycNE* in *Lactobacillus rhamnosus*, which leads to augmented indole-3-carboxaldehyde (I3A) production. Consequently, increased I3A leads to enhanced expression of IL-22, a cytokine known to alleviate inflammatory responses, by activating the AHR signaling pathway and triggering antimicrobial immunity, as well as tissue repair in barrier surfaces (29).

### 7.2. Shape the gut microbiota

Ginger has traditionally been used for regulating intestinal microflora and maintaining intestinal homeostasis in individuals with gastrointestinal disorders (104). A study suggest that orally administered GELNs may be involved in the communication between host gut cells and gut microbiota (25). Intestinal macrophages and epithelial cells can take up GELNs. GELNs promote intestinal epithelial cell proliferation and stimulate the expression of adhesion junction proteins (E-cadherin and desmocollin) in the colon epithelial cells of colitis mice (25). A similar study showed that GELNs taken by bacteria within the intestinal tract of mice preserve the balance of intestinal microbiota. GELNs carrying mdo-miR7267-3p targeted *Lactobacillus rhamnosus* monooxygenase ycnE, exhibited the ability to augment intestinal barrier function (29).

### 7.3. Direct antivirals

The ongoing COVID-19 pandemic is caused by SARS-CoV-2, a member of the coronaviridae family. Despite extensive research on COVID-19, there is currently no definitive treatment or fully effective vaccine (105). Three independent studies have suggested that miRNAs within GELNs could potentially slow the progression of COVID-19 (15, 41, 42). Kalarikkal et al. (15) found that Osa-miR-530-5p carried by GELNs indirectly inhibited *ORF1b* synthesis and suppressed SARS-CoV-2 replication by preventing ribosomal slip. Suresh et al. (41) detected the presence of specific miRNAs (miR-156a, miR-159, miR-5,077, miR-6,300, miR-166, and miR-5,059) within GELNs that were predicted to target SARS-CoV-2 genome. However, these two studies merely predicted the binding capabilities of miRNAs to the viral genome and lacked experimental evidence to validate these predictions. In contract, Teng et al. (42) demonstrated that two miRNAs (rlcv-miR-rL1-28-3p and alymiR396a-5p) in GELNs inhibit the SRAS-CoV-2 cytopathic effect in Vero E6 cells by suppressing the expression of the viral proteins S and Nsp12.

### 7.4. Drug delivery applications

Engineering EVs are one of the most commonly used drug delivery systems and are suitable as a co-delivery platform for chemotherapeutic drugs and nucleic acid agents (106). However, the use of EVs comes with several challenges for its application in clinical settings, including ethical concerns, safety risks, toxicity, and expensive production costs (107, 108). To address these challenges, researchers have widely adopted non-immunogenic and non-toxic PELNs due to their ease of large-scale production, and absence of ethical or zoonotic issues (78, 109, 110). Among PELNs, GELNs have been proven to be an outstanding carrier platform for efficient drug delivery (30, 46, 47, 49, 50, 57).

The primary goal of drug delivery systems is to minimize side effects while improving efficacy at lower drug doses (62). Doxorubicin (Dox) is a powerful anticancer agent, but its usage has been associated with numerous adverse effects, such as thrombocytopenia (111). Due to its slightly positive charge, Dox can interact with GELNs, which have a negative charge, to form bilayers and be encapsulated into GELNs through electrostatic interactions facilitated by sonication (47). Notably, Dox encapsulated in GELNs exhibited high loading efficiency, remarkable biocompatibility, and therapeutic effects against colon cancer (47). This is probably attributed to the increased localized dose of Dox in tumor tissues when GELNs is used for targeted delivery. Additionally, the release of Dox by GELNs occurs at the acidic pH of the tumor extracellular microenvironment, resulting in fewer drug side effects (47).

Unlike small molecules, siRNA is a relatively new addition to the therapeutic field and requires an efficient and specific delivery system (112). Presently, GELNs loaded with siRNA are being developed as a novel delivery method to treat various diseases, such as ulcerative colitis (46), cancer (30), and iron-loading disorder hereditary hemochromatosis (49). Specifically, GELNs loaded with siRNA-CD98 showed accurate targeting to colon tissues, which led to the suppression of CD98 expression, reduced colitis, and cancer-associated colitis (46). Specifically, The Bligh and Dyer method, similar to the preparation of liposomes, was used to obtain dry GELNs, which were then mixed with corresponding miRNAs and subjected to sonication to facilitate fusion (46). Similarly, Wang et al. (49) employed sonication-fused method to make GELNs carry with Dmt1 siRNA. Then, the Dmt1 siRNA in GELNs via oral administration to target genes implicated in iron-loading disorder hereditary hemochromatosis in mice, efficiently mitigating pre-existing iron overload (49). These findings demonstrated that GELNs are promising oral colon-targeted drug delivery systems (46). Another novel method was developed involving folate-conjugated arrowtail pRNA-3WJ to enhance the binding and targeting specificity of GELNs to KB cells (30). To enhance tumor-specific targeting of GELNs, they were incubated with arrow-tailed RNA. Then, I.V. administration of this engineered GELNs caused tumor growth inhibition in mice (30). However, the optimization of cargo-loading methods to achieve high loading efficacy in clinical practice remains challenging. Moreover, the mechanisms behind the stable behavior of GELNs in the physiological system remain unclear. The fact that the lipid bilayer structure of GELNs offers secure encapsulation of their cargo and protection against enzymatic degradation by proteinases and nucleases could be a reason (113). Additionally, a comprehensive protocol for GELNs preparation and characterization for colon-targeted siRNA delivery has been published in the BIO-PROTOCOL journal, which expands our understanding of the carrier capabilities of GELNs and their preparation (57).

## 8. Established clinical trials

So far, a few PELNs have entered the clinical trial stage. In 2011, the first clinical trial of PELNs for targeted delivery of curcumin to colon cancer tissues (NCT01294072) was conducted (114). Recently, a completed clinical trial of GELNs was conducted to evaluate the anti-inflammatory effects of GELNs on the gut lining of individuals with IBD, with and without curcumin supplementation (NCT04879810). Furthermore, a trial of GELNs investigating its potential to mitigate polycystic ovary syndrome (PCOS) was withdrawn before recruitment due to investigator departure (NCT03493984).

## 9. Perspectives and challenges

PELNs, a novel class of plant bioactive derivatives, possess numerous advantages such as non-toxic, low immunogenicity, good bioavailability, biocompatible, ease of absorption, cost-effective, environmentally friendly, and mass-produced platforms, in the prevention and treatment of diseases. These superior characteristics have been reviewed in many previous publications (12, 110, 115). Presently, PLENs research follows two avenues: one focuses on bioengineering application as a drug carrier; The other explores their direct pharmacological effects as biological agents, which is the primary subject of this review. We believe that these two directions are open and interrelated. Advancing materials and engineering developments will enhance the quality control systems for PELNs. Simultaneously, pharmacological activity analysis of PELNs will strengthen the evidence base for phytopharmaceuticals, expediting the development of complementary medicine.

Since the dawn of humankind, medicinal herbs have consistently supported our health in various forms. Processing techniques, such as drying and decoction, facilitates storage, transportation and preparation of herbs, but also lead to the loss of PELNs. In ancient, people directly chewed herbs to get therapeutic effects. We hypothesize that this process is accompanied by a large intake of PELNs. Meanwhile, ample evidence suggests that PELNs provide a tangible, characterizable form with multi-component and multi-target features (12). This consideration prompted our curiosity as to whether PELNs abundant in fresh herbs might yield novel or forgotten pharmacological effects. However, the special lipid structure of exosomes can alter the active substances enriched in HLENs, which makes the exploration of composition and temporal characteristics of drug release crucial. In addition, the developmental challenges of HELNs are similar to those of “daodi herb” (also known as authentic and superior medicinal herbal) (116). These challenges are mainly due to the seasonal and geographical factors of the plants. Future investigations, including pharmaceutical analysis, pharmacokinetics, pharmacotoxicology, and formulation development, should be performed to address these questions. Currently, however, researchers have not yet investigated this direction. In addition, we believe that further research is needed in the areas of the extraction, purification, physiochemical characterization, quality control, safety, and *in vivo* distribution of HELNs in order to reach the standards required for industrial and clinical applications.

Nevertheless, PELNs research remains in its developing stage and the concept of HELNs lacks exploration within this field. In light of the unique pharmacological activity of herbs, this review posits the new concept of HELNs and exemplifies their isolation, physical and biochemical characteristics, and application using GELNs. The objective of this review is to facilitate the development and application of HELNs, and future studies are encouraged to identify their additional properties, extending beyond GELNs.

There are still many challenges that need to be taken into consideration. (1) No standard method, like The Minimal Information for Studies of Extracellular Vesicles for MEVs (117, 118), has been established for the characterization and classification of HELNs. In addition, we strongly recommend that symbolic activity evaluation be incorporated into the quality control system for HELNs formulations, rather than just testing for particle size, morphology, and purity. (2) Extraction methods for HELNs need improvement, particularly for large-scale production, to minimize losses and reduce costs; (3) Investigation is needed to determine the best methods for maintaining the stability of HELNs during processing and storage. Borrowing cellular cryoprotectant or stabilizers may be an efficient shortcut for HELNs storage; (4) As HELNs contain unique and effective active ingredients, further research is necessary to explore their pharmacological properties.

## Author contributions

WH and HZ searched the literature, wrote and reviewed this manuscript, and provided funding support for this manuscript. All authors contributed to the article and approved the submitted version.

## Funding

This work was supported by the National Natural Science Foundation of China (82104302 and 82100931). The Scientific Research Foundation of Tongji Hospital (2021C15).

## Conflict of interest

The authors declare that the research was conducted in the absence of any commercial or financial relationships that could be construed as a potential conflict of interest.

## Publisher’s note

All claims expressed in this article are solely those of the authors and do not necessarily represent those of their affiliated organizations, or those of the publisher, the editors and the reviewers. Any product that may be evaluated in this article, or claim that may be made by its manufacturer, is not guaranteed or endorsed by the publisher.
